# The Closely Related CD103^+^ Dendritic Cells (DCs) and Lymphoid-Resident CD8^+^ DCs Differ in Their Inflammatory Functions

**DOI:** 10.1371/journal.pone.0091126

**Published:** 2014-03-17

**Authors:** Zhijun Jiao, Sammy Bedoui, Jamie L. Brady, Anne Walter, Michael Chopin, Emma M. Carrington, Robyn M. Sutherland, Stephen L. Nutt, Yuxia Zhang, Hyun-Ja Ko, Li Wu, Andrew M. Lew, Yifan Zhan

**Affiliations:** 1 The Walter and Eliza Hall Institute of Medical Research, Parkville, Victoria, Australia; 2 Key Laboratory of Medical Immunology & Department of Laboratory Medicine, Affiliated Hospital of Jiangsu University, Zhenjiang, China; 3 Department of Microbiology and Immunology, University of Melbourne, Parkville, Victoria, Australia; 4 Department of Medical Biology, University of Melbourne, Parkville, Victoria, Australia; 5 Tsignhua University and Peking University Joint Center for Life Sciences and Tsinghua University School of Medicine, Beijing, China; Université Libre de Bruxelles, Belgium

## Abstract

Migratory CD103^+^ and lymphoid-resident CD8^+^ dendritic cells (DCs) share many attributes, such as dependence on the same transcription factors, cross-presenting ability and expression of certain surface molecules, such that it has been proposed they belong to a common sub-lineage. The functional diversity of the two DC types is nevertheless incompletely understood. Here we reveal that upon skin infection with herpes simplex virus, migratory CD103^+^ DCs from draining lymph nodes were more potent at inducing Th17 cytokine production by CD4^+^ T cells than CD8^+^ DCs. This superior capacity to drive Th17 responses was also evident in CD103^+^ DCs from uninfected mice. Their differential potency to induce Th17 differentiation was reflected by higher production of IL-1β and IL-6 by CD103^+^ DCs compared with CD8^+^ DCs upon stimulation. The two types of DCs from isolated lymph nodes also differ in expression of certain pattern recognition receptors. Furthermore, elevated levels of GM-CSF, typical of those found in inflammation, substantially increased the pool size of CD103^+^ DCs in lymph nodes and skin. We argue that varied levels of GM-CSF may explain the contrasting reports regarding the positive role of GM-CSF in regulating development of CD103^+^ DCs. Together, we find that these two developmentally closely-related DC subsets display functional differences and that GM-CSF has differential effect on the two types of DCs.

## Introduction

DC subsets may differ in appearance, anatomical location, surface phenotype, use of transcription factors and function. However, migratory CD103^+^ DCs (hereafter called CD103^+^ DCs) and lymphoid-resident CD8^+^ DCs share some developmental, molecular and functional features. They require similar transcription factors for development, viz. IRF8, ID2 or Batf3 [1]–[4] and are the only DCs that express the chemokine receptors XCR1 [5], [6] and Clec9A [7]. CD8^+^ DCs can also express variable levels of CD103 [8]–[10] while both express little or no CD11b. On a functional level, both DC populations are able to cross-present antigens [11], [12] and can induce potent Th1 responses [13]–[18]. Consequently, some workers have proposed that the two types of DCs, despite their different tissue locations, are very closely related, if not identical [3], [6], [19]. However, there are several unanswered questions regarding the two closely-related DC subsets.

Do they function identically? As aforementioned, both DC populations are able to cross-present antigens [11], [12]. Recently, CD103^+^ DCs have been shown to be the candidate DCs to induce Th17 responses [18]. Although Th17 induction has not been reported to be a hallmark of CD8^+^ DCs, there has been no direct functional comparison between these subsets. Therefore, we decided to compare how potently the two DC subsets would differentiate T cells towards Th17 and what levels of Th17-promoting cytokines each produce.

Given that they are located differently, do they arm with the same pathogen-sensors? It has been shown previously that CD8^+^ DCs express higher levels of TLR3 than CD103^+^ DCs [20]. Recently, NLRC4 has been shown to play a preferential role in CD8^+^ DCs mediating IFN-γ production by memory CD8^+^ T cells [21]. It has been shown recently that an intrinsic defect in emigration from inflamed tissues by *Nlrp10−/−* migratory DCs caused defective T cell activation [22]. We therefore decided to determine whether CD103^+^ DCs and CD8^+^ DCs differ in expression of the pattern recognition receptors, particularly NLRPs.

GM-CSF as a versatile cytokine has profound effects on different DC types [23]. Are the two types of DCs influenced similarly by GM-CSF? GM-CSF is dispensable for development of CD8^+^ DCs [10], [24], [25]. In contrast, two reports found a positive role for GM-CSF on CD103^+^ DC development [18], [26], although another did not [27]. We therefore attempted to clarify this controversy by examining the effects of GM-CSF at physiological levels or pathological (elevated) levels.

Collectively, we demonstrated that two closely-related DC subsets differ in ability to differentiate Th cells, differ in expression of inflammasome components, and differ in GM-CSF requirement. These differences may lead to division of labor in response to different environmental cues.

## Materials and Methods

### Mice

C57BL/6 mice, GM-CSF knockout (GMKO) mice [28], GM-CSF transgenic (GMtg) mice on C57BL/6 mixed background [29] and littermate control mice, OVA specific CD4 TCR transgenic OT-II mice [30] and OVA specific CD8 TCR transgenic OT-I mice [31] were generated and maintained in the animal facility of the Walter and Eliza Hall Institute of Medical Research. HSV-1-specific CD4 TCR transgenic gDT-II mice [12], CD103−/− mice [32], Langerin-EGFP mice [33] were bred and maintained at the Department of Microbiology and Immunology, University of Melbourne. This study was approved by The Walter and Eliza Hall Institute's Animal Ethics Committee (#2011.015; #2013.015). All experiments with mice were conducted in accordance with the rules of The Walter and Eliza Hall Institute's Animal Ethics Committee and The Melbourne University Animal Ethics Committee.

### DC enrichment and flow cytometry

To compare migratory dermal CD103^+^ DCs with lymphoid-resident CD8^+^ DCs, peripheral lymph nodes (LNs) (viz. inguinal, axillary, brachial and superficial cervical) were harvested; mesenteric LNs and hence gut-derived CD11b^+^CD103^+^ DCs were excluded. LNs or spleens were digested for 20 min at room temperature with collagenase-DNase and then treated for 5 min with EDTA to disrupt T cell-DC complexes. Light density cells (1.080 g/cm^3^ osmolarity for LN cells, 1.077 g/cm^3^ osmolarity for spleen cells) were separated by centrifugation in Nycodenz medium (Nycomed Pharma AS, Oslo, Norway). Cells after staining for cell surface markers were sorted on FACSaria (BD Biosciences, San Jose, CA).

For analysis, light density cells were incubated with rat anti-mouse FcγRII/FcγRIII monoclonal antibody (2.4G2) for 15 min at 4°C, to block non-specific binding of Ab, before staining with various combinations of mAb to CD11c (N418), CD11b (M1/70), I-A/I-E (2G9), CD8 (53.6.7), CD103 (M290), CD80 (16-10A1), CD86 (GL1), Mac-3 (M3/84), Ly6C (AL-21), CD49a (BD Biosciences), CD326 (BioLegend, San Diego, CA). CD45 (30-F11, BD Biosciences) was used to identify leukocytes in the single cell suspension of digested skin preparation. Data acquisition was performed on an LSR flow cytometer or FACSaria (BD Biosciences). Data were analyzed with Flow-jo software.

### Viral infection and isolation of DCs from HSV-1 infected mice

The wild-type parental HSV-1 strain KOS (HSV-1) was titrated on Vero cells. Unless stated otherwise, mice were infected with 1×10^6^ plaque-forming units of HSV-1 [12]. Brachial LNs were collected at 5 d post infection. Single suspensions were enriched for conventional DCs and used for sorting of LN DC subsets as previously described [12]. The purity of sorted subsets was >95%.

### Immunization with CFA

Mice were treated with subcutaneous injections (femoral regions) of emulsified CFA (0.1 ml) (Sigma, St Louis, MO) containing 2.5 mg/ml heat-killed *Mycobacterium tuberculosis* H37 RA (Difco, Detroit, MI). LNs were harvested 7 d later for DC preparation.

### Cytokine production after in vitro stimulation

Sorted DCs were cultured at 1–2×10^4^/well in 0.2 ml volume in U-bottom 96-well plates in the presence or absence of a single TLR agonist. The following panel of TLR agonists was used: CpG ODN 1826 (2 µg/ml) (Coley Pharmaceutical, Ottawa, Canada), Poly I:C (50 µg/ml, Invivogen, San Diego, CA) and LPS (1 mg/ml) (Sigma, St Louis, MO). DCs were cultured for 24–36 h before supernatants were collected and analyzed for cytokine content using Bio-Plex cytokine kits according to manufacturer's instructions (Bio-Rad, Hercules, CA) [17].

### In vitro proliferative responses and cytokine production of antigen-specific T cells

Purified CD4^+^ OT-II or CD8^+^ OT-I cells and gDT-II CD4^+^ cells (50,000) were labeled with CellTrace violet or CFSE (Invitrogen) and cultured together with purified DC (10,000–20,000) with or without soluble OVA. Replicate cultures were in 200 µl medium in the U-bottom wells of 96-well plates. Where indicated, 2 ng/ml murine GM-CSF was included in cultures. Culture was normally for 3 d at 37°C in a humidified 10% CO2-in-air incubator. Proliferation of T cells was assessed by reduction in dye intensity of harvested cells. Harvested supernatants were assayed for cytokines by Bio-Plex (Bio-Rad).

### RT-PCR detection of gene expression

Total RNA was isolated from CD8^+^ DC (>95% pure) and CD103^+^ DC (>98% pure) using TRI Reagent (Ambion, Life Tecnologies, Mulgrave, Australia). Co-Precipitant Pink (Bioline, Alexandria, Australia) was added prior to alcohol precipitation to aid RNA recovery. cDNA was prepared using iScript Reverse Transcription Supermix (Bio-Rad). Real-time PCR was performed using SsoAdvanced SYBR Green Supermix (Bio-Rad) and gene-specific primers (300 nM; Sigma) on a CFX384 real-time PCR detection system (Bio-Rad). The specificity of each candidate PCR amplicon was evaluated by melting curve analysis. Analysis was performed using the CFX Manager Software version 3.0 (Bio-Rad) using multiple reference genes (18s, HPRT and Ubiquitin B).

All technical steps were performed according to The Minimum Information for Publication of Quantitative Real-Time PCR Experiments (MIQE) guidelines. Gene-specific primers were used as listed (**Table S1**) [34]. Controls consisting of ddH_2_O were negative for target and reference genes. The lengths of amplicons were between 69 and 311 bp. The efficiency-corrected quantification was performed automatically by the CFX Manager Software V3.0 (Bio-Rad) based on relative standard curves describing the PCR efficiencies of the target and the reference genes. CFX Manager Software V3.0 then calculated normalized expression using three reference target genes for each given sample. Expression stabilities of the reference genes were evaluated by the M value. The M value for each reference gene is the average pairwise variation for that gene with all the other tested genes. M value for heterogeneous samples was within the recommended range of <1.

**Statistics:** Data were analysed with Prism software. Mean and SEM were used to present the data. Two tail Student's T test for unpaired samples was used to evaluate differences between groups.

## Results

### CD103^+^ DCs and CD8^+^ DCs differentially express costimulatory molecules and pattern recognition receptors

The similarity between resident CD8^+^ DCs and migratory CD103^+^ DCs are well documented [3], [6], [19], but as outlined above there are indications of functional differences. Here we aimed to explore such differences.

**Figure 1:**
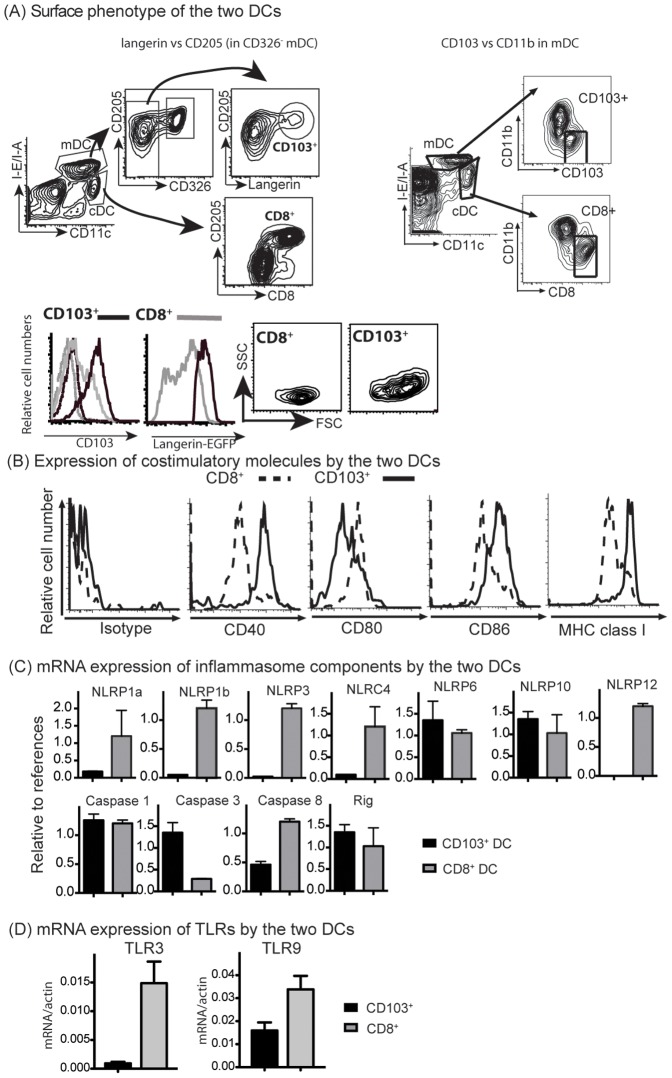
CD103^+^ DCs and CD8^+^ DCs differ in expression of costimulatory molecules, inflammasomes and TLR. (**A**) Cells of pooled cutaneous LNs from Langerin-EGFP mice and Langerin-EGFP/*CD103−/−* mice were analyzed. CD103^+^ DCs were identified as CD326^−^CD205^+^langerin^+^ within migratory DCs (mDC, CD11c^int^MHC II^high^); CD8^+^ DCs were identified as CD205^+^ CD8^+^ within cDCs (CD11c^high^MHC II^int^). Histograms show the expression of CD103 and langerin-EGFP by CD8^+^ and CD103^+^ DCs. For CD103 expression, CD8^+^ DCs (grey dot line) and CD205^+^CD11b^−^ migratory DCs (equivalent of CD103^+^ DCs, black dot line) from CD103−/− mice were included. (B) CD8^+^ and CD103^+^ DCs from B6 mice were analyzed for the expression of costimulatory molecules. (C&D) CD8^+^ and CD103^+^ DCs from B6 mice were sorted. RT-qPCR was performed for the indicated transcripts with 3 reference genes as controls. One of three repeated experiments is shown.

The definition of CD8^+^ DCs is straightforward. Conversely, the definition of migratory CD103^+^ DCs is more difficult due to heterogeneity of expression between CD103 and langerin in skin lymph nodes [35]. Furthermore, CD103 expression by resident CD8^+^ DCs and migratory CD103^+^ DCs is influenced by cell-extrinsic factors [10], [27]. Here we analyzed migratory CD103^+^ DCs in two ways. Firstly, we employed langerin-EGFP mice for analysis so that CD103^+^ DCs can be identified as migratory CD11c^int^MHC class II^high^CD205^+^CD326^−^CD11b^low/−^langerin^+^ DCs. Indeed, all CD205^+^CD326^−^CD11b^low/−^langerin^+^ DCs express CD103 while only a small fraction of CD8^+^ DCs express CD103 (**Fig. 1A** **, left panel**). Similarly, CD103^+^ DCs express langerin while CD8^+^ DCs express lower and variable levels of langerin. Notably, CD103^+^ DCs were larger (higher FSC) and more granular (higher SSC) than CD8^+^ DCs (**Fig. 1A** **, left panel**). For most of the study, CD103^+^ DCs were defined as CD11c^int^MHC class II^high^CD103^+^CD11b^low/−^ cells (**Fig. 1A** **, right panel**).

Relative to CD8^+^ DCs, CD103^+^ DCs generally expressed higher levels of CD40, CD86 and MHC class I. On the other hand, expression of CD80 was higher on CD8^+^ DCs than on CD103^+^ DCs (**Fig. 1B**).

In addition to these surface molecules relating to antigen presentation, we also examined expression of TLRs and NLRs by the two types of DCs. For this purpose, CD103^+^ DC and CD8^+^ DCs were sorted from peripheral LNs (purity >95%) and RNA was extracted for evaluation of the expression of inflammasome components and selected TLRs. CD8^+^ DCs expressed higher levels of NLRP1a/b, NLRP3, NLRC4 and NLRP12 than CD103^+^ DCs (**Fig. 1C**). Both DC subsets appear to express substantial levels of NLRP6 and NLRP10. Similar analysis of TLR expression revealed that CD8^+^ DCs expressed more TLR3 and TLR9 than CD103^+^ DC (>15 fold, p<0.01; 2 fold, p<0.05 respectively) (**Fig. 1D**).

Dermal CD103^+^ DCs that have migrated to the lymph nodes (LNs) expressed higher levels of MHC class II, certain costimulatory molecules and pattern-recognition molecules and therefore may be considered to be “more mature” than resting CD8^+^ DCs. For example, NLRP3 is upregulated by NF-κB activation [36]. Nevertheless, no uniformly higher expression of pattern-recognition molecules and costimulatory molecules by CD103^+^ DCs suggests that differential expression of these molecules by the two DC subsets may be not be totally due to maturation.

### Steady-state CD103^+^ DCs and CD8^+^ DCs induce naïve T cells to produce different cytokines

**Figure 2:**
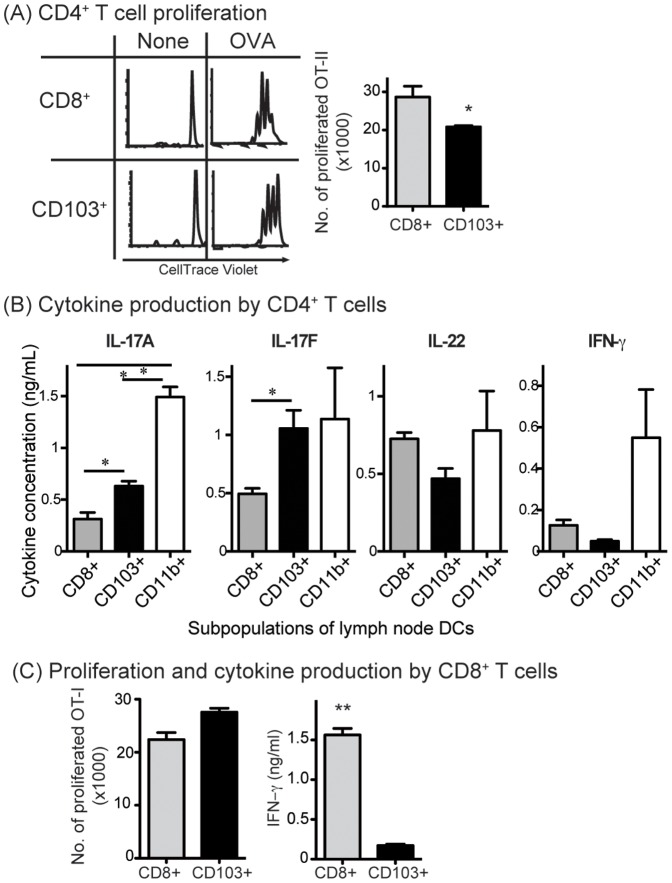
CD103^+^ DCs and CD8^+^ DCs induce naïve T cells to produce different cytokines. DC subsets from peripheral LNs of B6 mice were sorted. (A&B) DCs (10^4^/well in triplicate) were cultured with CellTrace violet-labeled OT-II cells (5×10^4^/well) with or without 1 mg/mL OVA for 3 d. Cell proliferation and supernatant cytokine levels were determined. (A) CD4^+^ T cell proliferation; (B) Cytokine production. Histograms show proliferation profile of OT-II, Bar graph shows mean ± SEM (*P<0.05, **P<0.01; Two tail Student's T test). (C) DCs (10^4^/well in triplicate) were cultured with CellTrace violet-labeled OT-I cells (5×10^4^/well) with or without 0.1 mg/mL OVA for 3 d. Cell proliferation and supernatant cytokine levels were determined. Bar graph shows mean ± SEM (**P<0.01; Two tail Student's T test).

Next, we compared the ability of CD103^+^ DCs and CD8^+^ DCs to induce T cell differentiation. Purified DCs were cultured with naïve OVA-specific TCR transgenic CD4 T cells (OT-II) with or without OVA protein for 3 d. CD103^+^ DCs stimulated higher amounts of IL-17 by OT-II cells, than did CD8^+^ DCs (**Fig. 2B**). Despite higher class II expression, CD103^+^ DCs stimulated a slightly weaker proliferative response in OT-II cells (**Fig. 2A**). In contrast to IL-17, CD103^+^ DCs stimulated reduced amounts of IFN-γ and IL-22 than did CD8^+^ DCs (**Fig. 2B**).

While both CD8^+^ DCs and CD103^+^ DCs are able cross-present antigens to CD8^+^ T cells [11], [12], [37], [38], a question remains as to whether effector function of CD8^+^ T cells is differentially activated by the two types of DCs. Here, we compared the two DC types for their ability to stimulate IFN-γ by CD8^+^ T cells. In repeated experiments, CD8^+^ DCs stimulated more OT-I-mediated IFN-γ production than CD103^+^ DCs, although both stimulated comparable proliferative responses (**Fig. 2C**). Production of IL-4, IL-5, IL-17 and IL-22 by OT-I cells, either with CD8^+^ DCs or CD103^+^ DCs was minimal (data not shown).

We also had isolated CD11b^+^ DC from skin-draining lymph node for functional evaluation. CD11b^+^ DCs in general were proficient to stimulate Th1/Th17 cytokine production (**Fig. 2B**). Compared to CD11b^+^ DCs, CD103^+^ DCs could produce comparable IL-17F, about half as much IL-17A and very low level of IFN-γ. Therefore, the contribution of CD103^+^ DCs to Th differentiation is substantial. However, since LN CD11b^+^ DCs contain multiple subsets (lymphoid-resident CD11b^+^ DCs, monocyte-derived CD11b^+^ DCs and dermal migratory CD11b^+^ DCs), the contribution of each CD11b^+^ subset to Th differentiation needs to be evaluated separately.

As CD103^+^ DCs originate from peripheral tissues like skin, we also attempted to evaluate the functional property of skin CD103^+^ DCs. Around 200–500 CD103^+^ DCs were recovered from trunk skin of a mouse. Due to low cell numbers recovered, no optimal proliferative response of OT-II T cells was detected with skin CD103^+^ DCs (data not shown).

### After viral infection, CD103^+^ DCs and CD8^+^ DCs induce naïve T cells to produce different cytokines

**Figure 3:**
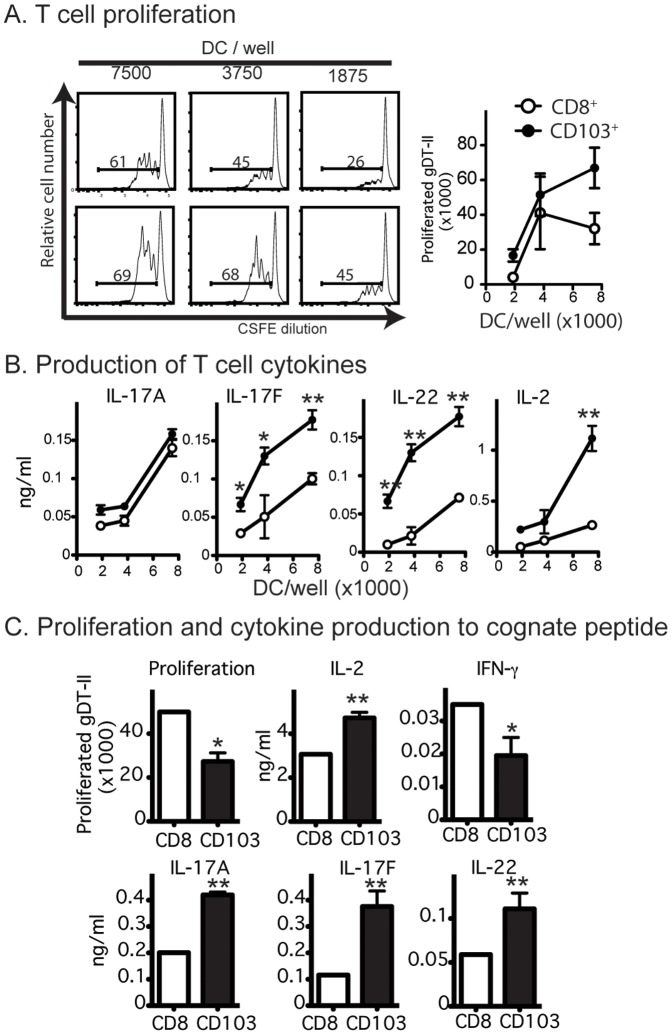
CD103^+^ DCs and CD8^+^ DCs have differential capacity to induce Th17 upon viral infection. (A) Proliferation of 5×10^4^ CFSE-labeled HSV-specific CD4^+^ T cells (gDT-II) after 60 h of culture together with serial dilutions of DC subsets isolated from brachial lymph nodes of mice infected withn HSV 5 d earlier. (B) Culture supernatants from (A) were measured for the indicated cytokines. (C) Proliferation and cytokine production with isolated DCs and exogenous source of antigenic peptide. Data (mean ± SEM) are one of two individual experiments. (**P<0.01; Two tailed Student's T test).

Previous work had shown that both CD103^+^ DCs and CD8^+^ DCs could capture viral antigens in vivo for class I and class II presentation [12]. However, Th17 differentiation was not examined. Here, the two types of DCs were isolated from axillary lymph nodes 5 d after herpes simplex virus (HSV) flank infection. We tested their capacity to induce proliferation and cytokine production by CFSE-labeled HSV-1-specific CD4^+^ T cells (gDT-II). As previously described [12], CD103^+^ DCs induced a stronger proliferative response than CD8^+^ DCs (**Fig. 3A**) and elicited more IL-17F and IL-22 (**Fig. 3B**).

The increased cytokine production induced by CD103^+^ DCs may have been a consequence of the differential antigen loading between the two subsets due to the site of infection. To account for this, we added saturating amounts of antigenic peptide and found that CD103^+^ DCs stimulated weaker gDT-II proliferation compared to CD8^+^ DCs (**Fig. 3C**). Despite this, CD103^+^ DCs induced higher production of Th17 cytokines (IL-17A, IL-17F and IL-22) by gDT-II cells (**Fig. 3C**). Thus, CD103^+^ DCs were more potent at inducing Th17 differentiation.

### CD103^+^ DCs and CD8^+^ DCs produce different cytokines and chemokines

**Figure 4:**
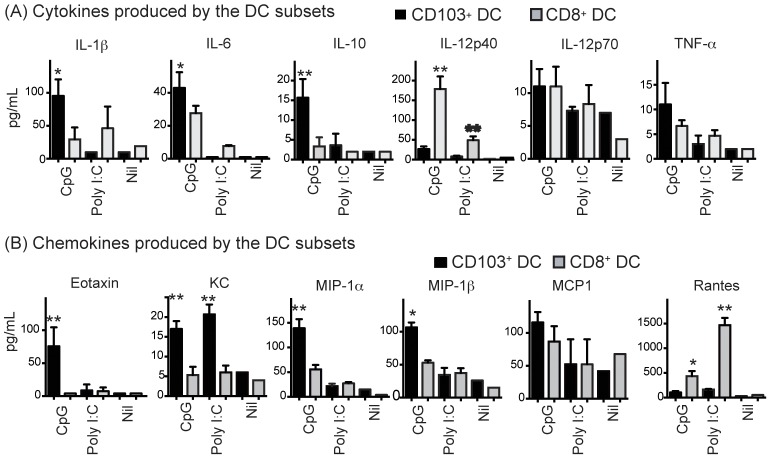
CD103^+^ DCs and CD8^+^ DCs produce different cytokines and chemokines. DCs were sorted from B6 mice and cultured at 5000 DCs per well in 96-well plates with (CpG or Poly I:C) or without (Nil) stimuli for 24 h. Exogenous GM-CSF (2 ng/mL) was included in cultures. Culture supernatants were assayed for cytokines (A) and chemokines (B). Data (mean ± SEM) are one of three individual experiments (*P<0.05, **P<0.01; Two tailed Student's T test).

To investigate the mechanism by which CD103^+^ DCs induce Th17 cells, we compared the production of cytokines and chemokines in response to TLR stimulation. Several cytokines known to be critical for Th differentiation were selected for evaluation including IL-1α, IL-1β, IL-6, IL-10, IL-12, IL-23 and TNF-α. We consistently observed that compared to CD8^+^ DCs, CD103^+^ DCs produced significantly higher levels of IL-1β, IL-6 and IL-10 upon activation by a TLR9 ligand, (**Fig. 4A**), despite expressing a lower level of TLR9 (**Fig. 1**). Low levels of IL-23 protein were produced by both types of DCs (data not shown). We also examined the production of several chemokines by the two types of DCs. In response to CpG, CD103^+^ produced more eotaxin, KC, MIP-1α and MIP-1β than CD8^+^ DCs (**Fig. 4B**). On the other hand, CD8^+^ DCs produced more RANTES (**Fig. 4B**). Collectively, our findings indicate that CD103^+^ DCs, relative to CD8^+^ DCs, produced more cytokines that favor Th17 differentiation.

### Elevated GM-CSF increases the numbers of CD103^+^ DCs

Controversy exists as to whether GM-CSF affects the development of CD103^+^ DCs per se [18], [26], or simply the expression of CD103 [27]. Here we explored further the role of GM-CSF in CD103^+^ DC development in mice deficient in GM-CSF and mice with GM-CSF over-expression.

In GM-CSF knockout (GMKO) mice, LN CD103^+^ DCs were proportionally reduced based on CD103 expression (**Fig. S1A**). However, LN CD103^+^ DCs from the same mice based on intracellular langerin staining was not reduced, compared to wild type (WT) mice (**Fig. S1A**). Furthermore, when CD8^−^CD326^−^CD11b^low/−^CD205^+^langerin^+^ DCs from WT and GMKO mice were compared for CD103 expression, GMKO DCs had lower CD103 expression than WT DCs (**Fig. S1B**). These data support the findings of [27] showing that physiological level of GM-CSF can modulate CD103 expression of CD103^+^ DCs.

In addition, we also used CD24 as a GM-CSF-independent surrogate marker of CD103 DC. Data do show that CD24^+^CD11b^−^ migratory DCs were comparable between WT and GMKO mice, while CD103^+^ within such a cohort is significantly smaller in GMKO mice. Thus data reinforces that GM-CSF deficiency primarily influences CD103 levels (**Fig. S1C**).

**Figure 5:**
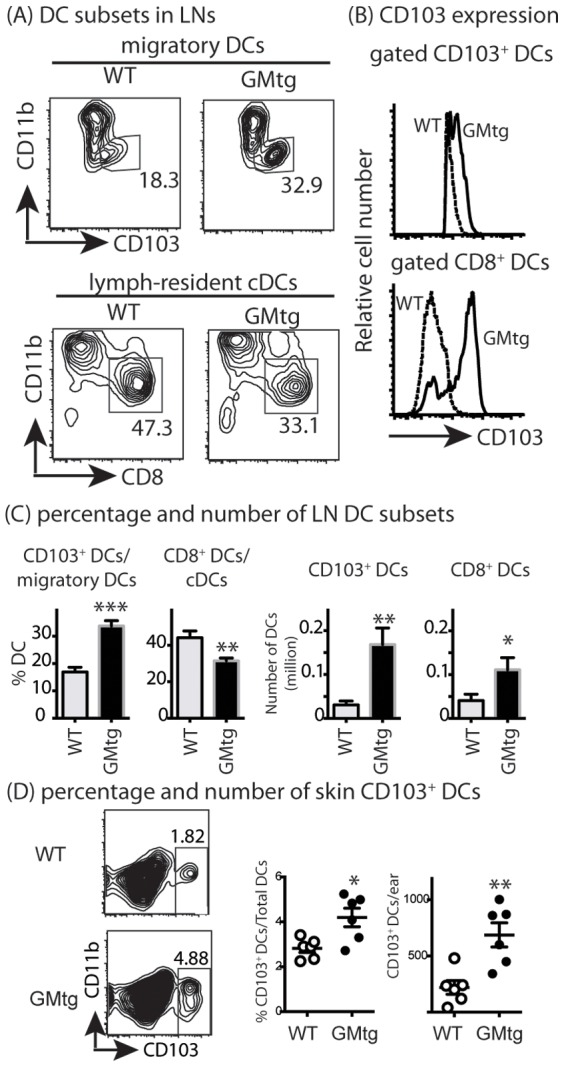
Elevated GM-CSF increase CD103^+^ DCs. DC-enriched LN cells were isolated from pooled LN for individual GMtg mice and wild type littermates. DC-enriched LN cells were stained for cell surface markers. (A) Gated CD11c^+^ cells were segregated into migratory DC (MHC class II^hi^ and CD11c^int^) and lymphoid-resident DC (MHC class II^int^ and CD11c^hi^) fractions. Numbers inside dot plots indicate percentage of gated populations. (B) CD103 expression by CD103^+^ DCs and CD8^+^ DCs is shown. (C) Mean numbers of DC subsets from cutaneous LNs of individual mice in each group are shown in bar graphs. Data are representative of >3 experiments. (D) Number of CD103^+^ DCs in ear skin. Each dot represents the data derived from an individual mouse. Horizontal line shows the mean ± SEM. Data are pooled from two experiments (*P<0.05, **P<0.01; Two tailed Student's T test).

Building on previous approaches, we evaluated CD103^+^ DCs in mice with elevated GM-CSF (to reflect what might occur during inflammation). In GM-CSF transgenic (GMtg) mice, the proportion of CD103^+^ DCs within migratory DCs (CD11c^int^I-A^high^) in pooled cutaneous lymph nodes was doubled (33.7±1.1% in GMtg vs 16.8±1.5 in WT; P<0.001) while the numbers of CD103^+^ DCs increased by 4-fold (168,000±21,000 vs 30,000±5,000 in WT, P<0.01) (**Fig. 5A & C**). In parallel, the percentage of CD8^+^ DCs within cDCs (CD11c^high^I-A^int^) was significantly lower in GMtg mice (31.5±0.9% vs 44.1±2.1 in WT; P<0.001). Due to a 2-fold increase in the number of total LN DCs, the absolute number of CD8^+^ DCs was higher in GMtg mice (111,000±16,000 vs 41,000±9,000 in WT; P<0.05) (**Fig. 5A & C**).

An increase in CD103^+^ DCs was also observed in ear skin (**Fig. 5D**). Within gated CD11c^+^MHC class II^+^ cells isolated from skin dermis, the percentage of CD103^+^ DCs was >1.5-fold higher in GMtg mice. The number of CD103^+^ DCs was >2-fold higher in GMtg mice (687±107 vs 220±61 in WT mice).

Whereas the increase in CD103 expression on CD8^+^ DCs from GMtg mice (compared with WT) was large (MFI increased 9-fold; p<0.001; **Fig. 5B**), the increase on migratory DCs from GMtg mice was modest (MFI increased 1.5-fold; p<0.01). Thus, elevated GM-CSF mainly increases the number of CD103^+^ DCs and modestly increases CD103 expression in CD103^+^ migratory DCs.

**Figure 6:**
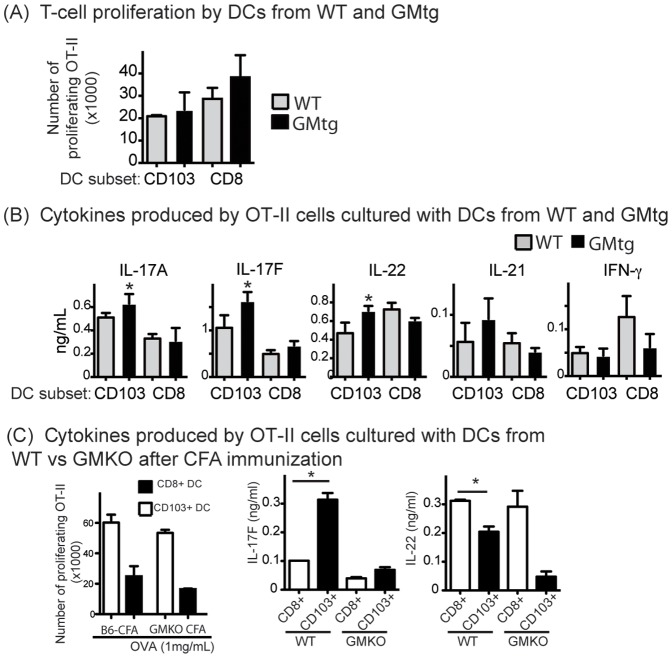
GM-CSF modulates the capacity of CD103^+^ DCs to induce Th differentiation. CD103^+^ DCs and CD8^+^ DCs were purified from LNs of GMtg and wild type littermates. DCs were cultured with CellTrace violet-labeled OT-II cells and 1 mg/ml OVA for 3 d. (A) Proliferation was determined by dye dilution. Bar graphs show numbers of proliferating OT-II cells from triplicate cultures. (B) Cytokines in culture supernatants were measured. Bar graph shows mean ± SEM (*P<0.05; Two tail Student's T test). >3 experiments were performed with similar results. (C) DCs (5×10^3^/well in triplicate) were cultured with CellTrace voilet-labelled OT-II cells (1×10^4^/well) for 3 d. Supernatants were collected for analysis of cytokine production. Bar graph shows mean ± SEM of cell proliferation and selected cytokines (*P<0.05; Two tailed Student's T test).

Several recent reports have revealed that GM-CSF enhanced cross-presentation by CD8^+^ DCs [10], [39], [40]. We next examined the impact of GM-CSF on the ability of the two DC subsets to induce T-cell differentiation. We first investigated the impact of elevated GM-CSF in vivo (GMtg mice) on the DC subsets. It should be added that exogenous GM-CSF (2 ng/ml) was added into all cultures to adjust for potential differences in GM-CSF production among the groups. For both CD103^+^ and CD8^+^ DCs, DCs from GMtg mice did not stimulate a significantly higher proliferative response than the same types of DCs from WT mice (**Fig. 6A**). On the other hand, CD103^+^ DCs from GMtg mice stimulated higher production of IL-17 and IL-22 by CD4^+^ T cells, compared to CD103^+^ DCs from WT mice (**Fig. 6B**). CD8^+^ DCs from both types of mice stimulated lower but comparable production of IL-17 by CD4^+^ T cells (**Fig. 6B**). We also observed that CD103^+^ DCs from CFA immunized mice stimulated OT-II cell to produce more IL-17F than did CD8^+^ DCs (**Fig. 6C**). GM-CSF deficiency reduced the potency of CD103^+^ DCs to prime T cells to produce IL-17F and IL-22 (**Fig. 6C**). Collectively, our findings indicate that GM-CSF seems not to enhance the ability of DCs to stimulate T-cell proliferation but does modestly enhance CD103^+^ DCs at inducing Th17 differentiation.

## Discussion

Migratory CD103^+^ DCs and lymphoid-resident CD8^+^ DCs share so many developmental and functional features that some have proposed that they belong as one subset [6], [19]. We examined the phenotypic and functional differences of these two closely related DC subsets from skin draining lymph nodes. We revealed that these two types of DCs differ in Th17 inducing ability, probably related to their differential ability to induce cytokines influencing Th differentiation. We showed here that with in vivo and in vitro antigen exposure, CD103^+^ DCs stimulated a stronger IL-17 production by CD4^+^ T cells. We found that CD103^+^ DCs, compared to CD8^+^ DCs, produced more IL-1 and IL-6, cytokines known to promote Th17 differentiation [41]. Furthermore, our study suggests that elevated GM-CSF enhanced the number as well as function of migratory CD103^+^ DCs.

In addition to cytokine production, CD103^+^ and CD8^+^ DCs also differed in expression of several NLRPs and TLRs. CD8^+^ DCs expressed higher levels of NLRP1a/b, NLRP3, NLRC4 and NLRP12 than CD103^+^ DCs, at least at the mRNA level. Fittingly to the expression pattern, NLRC4 in CD8^+^ DCs has recently been shown to mediate IFN-γ production by memory CD8^+^ T cells [21]. Although CD103^+^ DCs expressed lower levels of NLRP1a/b, NLRP3, NLRC4 and NLRP12, they did express high levels of NLRP6 and NLRP10. The higher NLRP10 expression is consistent with the Th17 promoting activity of CD103^+^ DCs, as *Nlrp10−/−* mice have markedly reduced IL-17 production [22].

Th17 cells can produce several cytokines including IL-17A, IL-17F, IL-22 and IL-21 [42]–[44]. We measured all four Th17 cytokines in our study. CD103^+^ DCs isolated from HSV-infected mice stimulated higher production of IL-17F and IL-22 than did CD8^+^ DCs, with the difference in production of IL-17A being less conspicuous (**Fig. 3**). Decoupling of IL-17A and IL-17F expression patterns has been noticed previously [45]. CD4^+^ T cells with reduced TCR-induced phospholipase C-γ activation expressed less IL-17A, while maintaining relatively normal expression of ROR-γT and IL-17F. Calcineurin inhibition also preferentially reduced IL-17A expression [45]. Functionally, IL-17A and IL-17F have overlapping yet distinct roles in host immune and defense mechanisms [46].

Functional differences between the two types of DCs were also reflected by their capacity to stimulate IFN-γ production by CD8^+^ T cells via cross-presentation. Although both CD8^+^ DCs and CD103^+^ DCs cross-present antigens to CD8^+^ T cells [11], [12] and stimulated a similar CD8^+^ T-cell proliferative response (**Fig. 1**), CD8^+^ DCs stimulated higher production of IFN-γ by CD8^+^ T cells (**Fig. 1**). Taken together our study demonstrates that the two seemingly closely related DC subsets nevertheless differ quite substantially in their ability to influence the differentiation of CD4^+^ T cells, a phenomenon that could be related to their differential expression of pattern recognition receptors and their disparate ability to produce Th17-inducing cytokines. Whether these differences represent cell-intrinsic functional specializations or rather reflect differences in their maturation status (migratory vs. LN-resident) will have to be resolved in future studies. Regardless of the cause of these differences, the present findings indicate that naïve CD4^+^ T cells entering a LN, in which both CD103 DC and CD8 DC present their cognate antigen on MHC II, will have differential differentiation fates depending on whether they are activated by CD103^+^ DC or CD8^+^ DC.

Numerous studies over the last two decades have established that GM-CSF is a pivotal cytokine in many inflammatory conditions [47]. GM-CSF-influenced inflammation is also critically mediated by Th17 cells [48], [49]. Several recent publications suggest that GM-CSF derived from Th17 cells is critical for inflammation and pathology [50]–[52]. It was also reported that GM-CSF was critical for Th17 induction [18], [27], [53]. We contend that CD103^+^ DCs are likely one of the subsets whose Th17-inducing capacity is modulated by GM-CSF.

The regulation of the pool size of CD103^+^ DCs by GM-CSF remains incompletely understood. Two studies showed that CD103^+^ DCs were reduced in GM-CSF or GM-CSF receptor deficient mice [18], [26], while a third study concluded that GM-CSF is dispensable for the development of CD103^+^ DCs [27]. Edelson et al showed that CD103 expression on CD103^+^ DCs was lower in GM-CSFR deficient mice than in WT mice. Thus, they surmised that reduction in CD103^+^ DCs reflects the lower expression on CD103^+^ (identified as CD326^int^ langerin^+^) DCs, rather than a “loss” of the population. Notably, definition of CD103^+^ cells is less straightforward. It has been reported that CD103^+^ DCs contains langerin^+^ and langerin^−^ subsets while langerin^−^ cells contained CD103^+^ cells [35]. Given that GM-CSF influences CD103 expression [27], how to accurately assess the CD103^+^ population remains to be fully resolved.

Nevertheless, based on our findings with elevated GM-CSF, we suggest that GM-CSF indeed acts a positive regulator for CD103^+^ DCs, albeit the development of CD103^+^ DCs can still occur in its absence. With elevated GM-CSF, the numbers of CD103^+^ DCs increased significantly in both peripheral LNs and tissues. We reason that the discrepancy regarding the role of GM-CSF in development of CD103^+^ DCs can be partially explained by levels of GM-CSF. GM-CSF is an ”inflammatory” cytokine and is at very low levels in the steady state. Thus in a “clean” environment, GM-CSF levels may be too low to have any impact. In a “dirty” housing environment or iatrogenically induced infection/inflammation, the role of GM-CSF may become clearer. An example of the influence of GM-CSF levels is the regulation of CD103 on CD8^+^ DCs [10] (also herein). The proportion of spleen CD8^+^ DCs expressing appreciable levels of CD103 can be as low as 5–10% in the steady state, which is only marginally higher than that found in GM-CSF deficient mice. However, during infection, the majority of CD8^+^ DCs from WT mice become CD103^high^, whereas CD8^+^ DCs from GM-CSF deficient mice remain CD103^low^ [10].

How GM-CSF regulates CD103^+^ DCs is incompletely understood. One recent suggestion is that GM-CSF regulates their survival since GM-CSF-unresponsive CD103^+^ DCs lose mitochondrial integrity [26]. However, it should be pointed out that CD103^−^ dermal DCs from the same mice showed similar mitochondrial changes, even though the pool size of CD103^−^ dermal DCs was not grossly affected by GM-CSF deficiency [26].

Apart from regulating cell number, we also found that GM-CSF also regulates the function of CD103^+^ DCs. Elevated levels of GM-CSF increased the ability of CD103^+^ DCs to secrete IL-1β and IL-6 and to induce Th17 differentiation. Therefore, GM-CSF shows differential effects on the two types of DCs viz. it promotes the generation of CD103^+^ DCs but not CD8^+^ DCs and strongly enhances CD103 expression on CD8^+^ DCs but not on CD103^+^ DCs.

Taken together, our study has revealed that the two seemingly closely related DCs have a different propensity in regulating Th17 differentiation. For both function and numbers, the two DC types also have a differential dependence on GM-CSF. Our findings highlight key differences between the two DC subsets and we would predict that the two types of DCs play distinct roles in T cell differentiation and pathogenesis in a GM-CSF mediated inflammation.

## Supporting Information

Figure S1 GM-CSF defciency reduced CD103 expression of CD103^+^ DCs. DC enriched LN cells were isolated from pooled LN of GMKO mice and WT mice. DC-enriched LN cells were then stained for cell surface markers and intracellular molecule langerin. (A) Gated CD8^−^CD326^−^CD11c^+^ cells were analyzed for expression of the indicated markers. Number inside dot plots indicates percentage of gated populations. (B). Histogram shows CD103 expression by CD8^+^ DCs (grey) and CD103^+^ DCs (black) from GMKO (dashed line) and WT mice (solid line). (C) Gated CD8^−^CD11b migratory DC (migDC) were analyzed for CD24 and CD103 expression. Bar graphs show % of CD24^+^ DC within CD8^−^ migDCs (left) and CD24^+^CD103^+^ within CD24^+^CD11b^−^ mig DCs (right). P value was calculated by two tailed Student's T test.
(TIF)

Table S1 PCR primers and PCR efficiency.
(DOCX)
