# Supplementary figures and images for: The Closely Related CD103+ Dendritic Cells (DCs) and Lymphoid-Resident CD8+ DCs Differ in Their Inflammatory Functions
Source: PLoS One. 2014 Mar 17;9(3):e91126. doi: 10.1371/journal.pone.0091126 (PMC3956455; doi:10.1371/journal.pone.0091126)

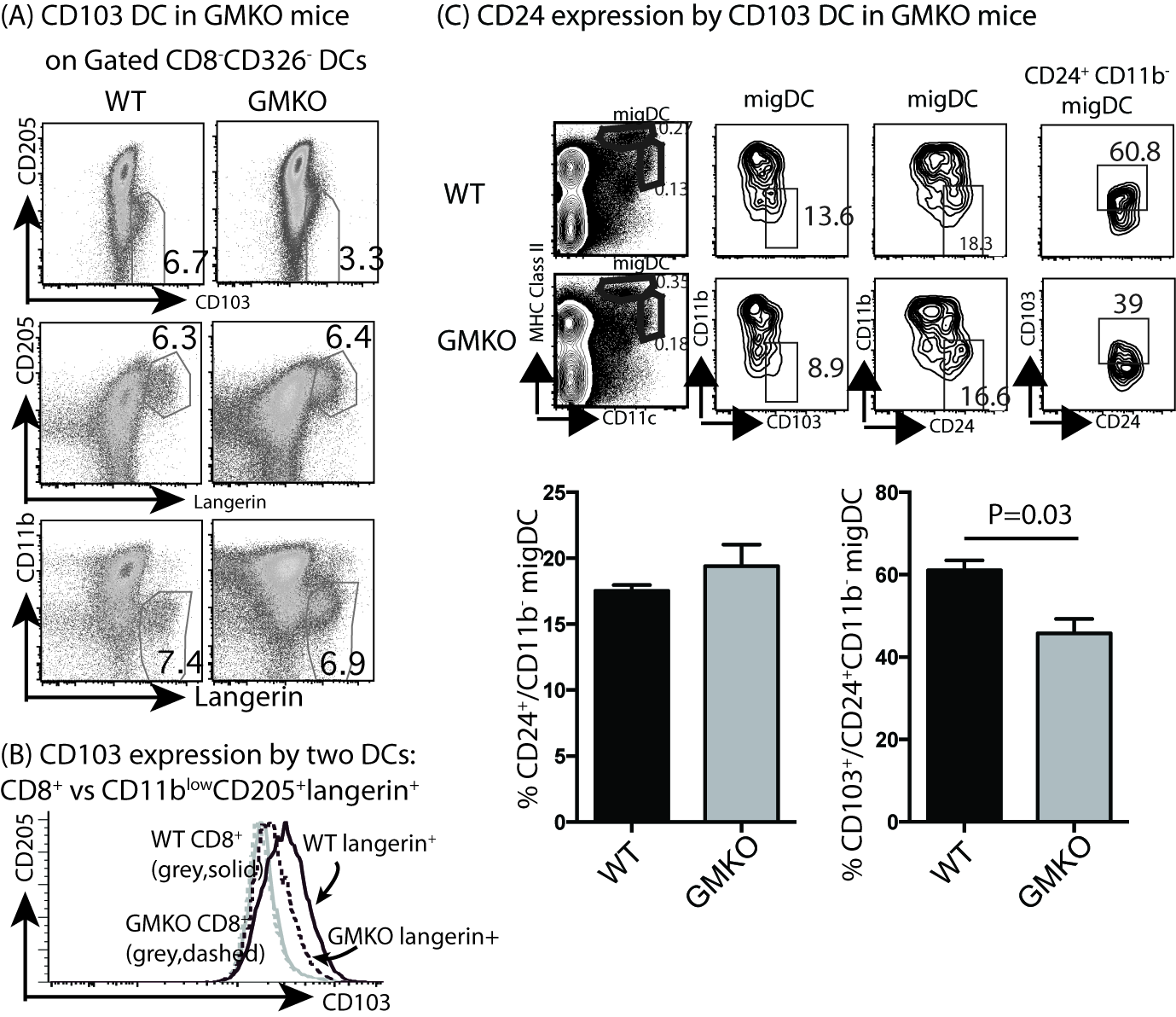

Supplement: Figure S1 — GM-CSF defciency reduced CD103 expression of CD103+ DCs. DC enriched LN cells were isolated from pooled LN of GMKO mice and WT mice. DC-enriched LN cells were then stained for cell surface markers and intracellular molecule langerin. (A) Gated CD8−CD326−CD11c+ cells were analyzed for expression of the indicated markers. Number inside dot plots indicates percentage of gated populations. (B). Histogram shows CD103 expression by CD8+ DCs (grey) and CD103+ DCs (black) from GMKO (dashed line) and WT mice (solid line). (C) Gated CD8−CD11b migratory DC (migDC) were analyzed for CD24 and CD103 expression. Bar graphs show % of CD24+ DC within CD8− migDCs (left) and CD24+CD103+ within CD24+CD11b− mig DCs (right). P value was calculated by two tailed Student's T test. (TIF) [file pone.0091126.s001.tif]
