# Supplementary material for: The Closely Related CD103+ Dendritic Cells (DCs) and Lymphoid-Resident CD8+ DCs Differ in Their Inflammatory Functions
Source: PLoS One. 2014 Mar 17;9(3):e91126. doi: 10.1371/journal.pone.0091126 (PMC3956455; doi:10.1371/journal.pone.0091126)
Supplement: Table S1 — PCR primers and PCR efficiency. (DOCX) [file pone.0091126.s002.docx]

**Table S1. PCR primers and PCR efficiency**

| Gene | Product size | Accession number | Primer sequence | Efficiency |
| --- | --- | --- | --- | --- |
| NLRP1a | 108 bp | NM_001004142 | Forward: 5'-ATGTGGACCCAACCTTCAAA |  |
|  |  |  | Reverse: 5'-GTACGTGCTCCTGGAAAGGT | 107.05% |
| NLRP1b | 116 bp | NM_001040696 | Forward: 5'-CCCAGCACAAGACTCCACT |  |
|  | 95 bp | NM_001162414 | Reverse: 5'-CCAACCACCATGTGACTCTG | 107.25% |
| NLRP2 | 108 bp | NM_177690 | Forward: 5'-GACGTCCTGAGAAAGCTGGA |  |
|  |  |  | Reverse: 5'-GGTCCATTGTCAGCTTGTTG | 94.65 |
| NLRP3 | 101 bp | NM_145827 | Forward: 5'-CCTTGGACCAGGTTCAGTGT |  |
|  |  |  | Reverse: 5'-AGAAGAGACCACGGCAGAAG | 104.20% |
| NLRC4 | 112 bp | NM_001033367 | Forward: 5'-GACGCTTTGACTCACCACAA |  |
|  |  |  | Reverse: 5'-CGTTCAATGCAAAGAGGTCA | 98.50% |
| NLRP6 | 118 bp | NM_001081389 | Forward: 5'-GCAGACGAGCTGCCTACTTT |  |
|  |  |  | Reverse: 5'-GCTCCTGGTAACAGCTCCTG | 101.75% |
| NLRP10 | 108 bp | NM_175532 | Forward: 5'-GACCTTGAGATCTGGCGTTG |  |
|  |  |  | Reverse: 5'-AGGTCATTCAAGGCCCAAAG | 108% |
| NLRP12 | 111 bp | NM_001033431 | Forward: 5'-CCTCTTTGAGCCAGACGAAG |  |
|  |  |  | Reverse: 5'-GCCCAGTCCAACATCACTTT | 107.30% |
| Hprt1 | 311 bp | NM_013556 | Forward: 5'-GGGGGCTATAAGTTCTTTGC |  |
|  |  |  | Reverse: 5'-TCCAACACTTCGAGAGGTCC | 98.90% |
| UbiquitinB | 69 bp | NM_011664 | Forward: 5'-TGGCTATTAATTATTCGGTCTGCAT |  |
|  |  |  | Reverse: 5'-GCAAGTGGCTAGAGTGCAGAGTAA | 99.70% |
| 18s | 124 bp | NR_003278 | Forward: 5'-GCAATTATTCCCCATGAACG |  |
|  |  |  | Reverse: 5'-AGGGCCTCACTAAACCATCC | 107.37% |
